# Supplementary material for: Network analysis of the human structural connectome including the brainstem
Source: PLoS One. 2023 Apr 6;18(4):e0272688. doi: 10.1371/journal.pone.0272688 (PMC10079027; doi:10.1371/journal.pone.0272688)
Supplement: S1 Table — (PDF) [file pone.0272688.s001.pdf]

ID	Brain structure	Number of streamlines connected to the structure	Structure volume (mm^3)
1	Left-Lateral-Ventricle	1190	6443.281
2	Left-Inf-Lat-Vent	45	235.092
3	Left-Cerebellum-Cortex	3939	57968.859
4	Left-Thalamus-Proper	4670	8619.509
5	Left-Caudate	2377	3851.667
6	Left-Putamen	4204	5540.322
7	Left-Pallidum	1552	1359.246
8	3rd-Ventricle	348	782.034
9	4th-Ventricle	434	1825.385
10	Brain-Stem	13150	22167.976
11	Left-Hippocampus	852	4564.249
12	Left-Amygdala	190	1593.61
13	CSF	362	1063.154
14	Left-Accumbens-area	384	573.736
15	Left-VentralDC	3784	4292.068
16	Left-vessel	102	59.189
17	Left-choroid-plexus	554	1125.245
18	Right-Lateral-Ventricle	978	5884.33
19	Right-Inf-Lat-Vent	38	232.622
20	Right-Cerebellum-Cortex	4659	59587.369
21	Right-Thalamus-Proper	4573	7521.443
22	Right-Caudate	2378	3952.693
23	Right-Putamen	4037	5609.062
24	Right-Pallidum	658	1493.601
25	Right-Hippocampus	985	4627.455
26	Right-Amygdala	226	1655.291
27	Right-Accumbens-area	435	600.805
28	Right-VentralDC	3927	4299.531
29	Right-vessel	148	71.884
30	Right-choroid-plexus	500	1235.485
31	Optic-Chiasm	264	231.912
32	CC_Posterior	231	960.037
33	CC_Mid_Posterior	316	478.638
34	CC_Central	436	514.796
35	CC_Mid_Anterior	417	515.937
36	CC_Anterior	342	900.829
37	ctx-lh-bankssts	192	2862.336
38	ctx-lh-caudalanteriorcingulate	557	3022.485
39	ctx-lh-caudalmiddlefrontal	1967	7065.912
40	ctx-lh-cuneus	467	2200.646
41	ctx-lh-entorhinal	268	865.957
42	ctx-lh-fusiform	762	7097.376
43	ctx-lh-inferiorparietal	1090	10072.529
44	ctx-lh-inferiortemporal	612	6603.035
45	ctx-lh-isthmuscingulate	927	3957.884
46	ctx-lh-lateraloccipital	816	8963.47

47	ctx-lh-lateralorbitofrontal	1029	6725.032
48	ctx-lh-lingual	773	5273.808
49	ctx-lh-medialorbitofrontal	1528	3930.844
50	ctx-lh-middletemporal	603	5530.654
51	ctx-lh-parahippocampal	299	1668.477
52	ctx-lh-paracentral	966	3732.204
53	ctx-lh-parsopercularis	1629	3869.169
54	ctx-lh-parsorbitalis	183	867.128
55	ctx-lh-parstriangularis	642	3038.14
56	ctx-lh-pericalcarine	482	3075.872
57	ctx-lh-postcentral	2426	7322.379
58	ctx-lh-posteriorcingulate	878	4473.498
59	ctx-lh-precentral	5905	13190.15
60	ctx-lh-precuneus	1658	9317.325
61	ctx-lh-rostralanteriorcingulate	500	2617.448
62	ctx-lh-rostralmiddlefrontal	1974	12890.373
63	ctx-lh-superiorfrontal	6817	18488.612
64	ctx-lh-superiorparietal	2264	12374.398
65	ctx-lh-superiortemporal	946	7718.647
66	ctx-lh-supramarginal	1003	8872.386
67	ctx-lh-frontalpole	134	190.543
68	ctx-lh-temporalpole	200	661.269
69	ctx-lh-transversetemporal	335	807.284
70	ctx-lh-insula	1159	8607.619
71	ctx-rh-bankssts	179	2950.619
72	ctx-rh-caudalanteriorcingulate	673	3172.229
73	ctx-rh-caudalmiddlefrontal	2115	6180.176
74	ctx-rh-cuneus	503	2191.445
75	ctx-rh-entorhinal	360	661.167
76	ctx-rh-fusiform	794	6957.369
77	ctx-rh-inferiorparietal	1265	12013.279
78	ctx-rh-inferiortemporal	629	6112.701
79	ctx-rh-isthmuscingulate	666	3573.922
80	ctx-rh-lateraloccipital	705	8876.928
81	ctx-rh-lateralorbitofrontal	1246	6661.849
82	ctx-rh-lingual	723	5407.399
83	ctx-rh-medialorbitofrontal	1317	3406.247
84	ctx-rh-middletemporal	807	6306.081
85	ctx-rh-parahippocampal	350	1680.065
86	ctx-rh-paracentral	1267	4657.647
87	ctx-rh-parsopercularis	1071	3563.056
88	ctx-rh-parsorbitalis	274	1191.086
89	ctx-rh-parstriangularis	651	3366.89
90	ctx-rh-pericalcarine	512	3168.029
91	ctx-rh-postcentral	2041	7181.13
92	ctx-rh-posteriorcingulate	883	4517.583
93	ctx-rh-precentral	5109	13710.037
94	ctx-rh-precuneus	1627	10019.167
95	ctx-rh-rostralanteriorcingulate	376	2153.274
96	ctx-rh-rostralmiddlefrontal	1827	13480.068

97	ctx-rh-superiorfrontal	6864	18600.449
98	ctx-rh-superiorparietal	2516	11960.449
99	ctx-rh-superiortemporal	1019	6898.772
100	ctx-rh-supramarginal	953	8860.182
101	ctx-rh-frontalpole	234	285.97
102	ctx-rh-temporalpole	279	613.389
103	ctx-rh-transversetemporal	258	576.932
104	ctx-rh-insula	1510	8912.524
