# Supplementary material for: Network analysis of the human structural connectome including the brainstem
Source: PLoS One. 2023 Apr 6;18(4):e0272688. doi: 10.1371/journal.pone.0272688 (PMC10079027; doi:10.1371/journal.pone.0272688)
Supplement: S2 Table — (PDF) [file pone.0272688.s002.pdf]

Brain structure	Number of streamlines connected to the structure
Left-Cerebellum-Cortex	529
Left-Thalamus-Proper	3818
Left-Caudate	1740
Left-Putamen	3239
Left-Pallidum	999
Left-Hippocampus	383
Left-Amygdala	74
Left-Accumbens-area	277
Right-Cerebellum-Cortex	490
Right-Thalamus-Proper	3129
Right-Caudate	1712
Right-Putamen	2791
Right-Pallidum	526
Right-Hippocampus	502
Right-Amygdala	88
Right-Accumbens-area	313
ctx-lh-bankssts	202
ctx-lh-caudalanteriorcingulate	532
ctx-lh-caudalmiddlefrontal	1649
ctx-lh-cuneus	390
ctx-lh-entorhinal	261
ctx-lh-fusiform	732
ctx-lh-inferiorparietal	994
ctx-lh-inferiortemporal	633
ctx-lh-isthmuscingulate	758
ctx-lh-lateraloccipital	639
ctx-lh-lateralorbitofrontal	898
ctx-lh-lingual	578
ctx-lh-medialorbitofrontal	1068
ctx-lh-middletemporal	598
ctx-lh-parahippocampal	256
ctx-lh-paracentral	425
ctx-lh-parsopercularis	1488
ctx-lh-parsorbitalis	162
ctx-lh-parstriangularis	564
ctx-lh-pericalcarine	399
ctx-lh-postcentral	1898
ctx-lh-posteriorcingulate	762
ctx-lh-precentral	3784
ctx-lh-precuneus	1089
ctx-lh-rostralanteriorcingulate	409
ctx-lh-rostralmiddlefrontal	1456
ctx-lh-superiorfrontal	3451
ctx-lh-superiorparietal	1612
ctx-lh-superiortemporal	878
ctx-lh-supramarginal	960
ctx-lh-frontalpole	89
ctx-lh-temporalpole	192

ctx-lh-transversetemporal	333
ctx-lh-insula	1110
ctx-rh-bankssts	179
ctx-rh-caudalanteriorcingulate	593
ctx-rh-caudalmiddlefrontal	1661
ctx-rh-cuneus	409
ctx-rh-entorhinal	315
ctx-rh-fusiform	748
ctx-rh-inferiorparietal	1156
ctx-rh-inferiortemporal	605
ctx-rh-isthmuscingulate	553
ctx-rh-lateraloccipital	570
ctx-rh-lateralorbitofrontal	956
ctx-rh-lingual	580
ctx-rh-medialorbitofrontal	849
ctx-rh-middletemporal	711
ctx-rh-parahippocampal	306
ctx-rh-paracentral	671
ctx-rh-parsopercularis	1010
ctx-rh-parsorbitalis	236
ctx-rh-parstriangularis	574
ctx-rh-pericalcarine	431
ctx-rh-postcentral	1559
ctx-rh-posteriorcingulate	780
ctx-rh-precentral	3178
ctx-rh-precuneus	1040
ctx-rh-rostralanteriorcingulate	290
ctx-rh-rostralmiddlefrontal	1306
ctx-rh-superiorfrontal	3154
ctx-rh-superiorparietal	1738
ctx-rh-superiortemporal	868
ctx-rh-supramarginal	878
ctx-rh-frontalpole	139
ctx-rh-temporalpole	238
ctx-rh-transversetemporal	256
ctx-rh-insula	1322
