# Supplementary material for: Network analysis of the human structural connectome including the brainstem
Source: PLoS One. 2023 Apr 6;18(4):e0272688. doi: 10.1371/journal.pone.0272688 (PMC10079027; doi:10.1371/journal.pone.0272688)
Supplement: S3 Table — This matrix corresponds with the standard MRtrix parcellation scheme, widely used in studies of brain networks. The list of cortical structures remained the same. (PDF) [file pone.0272688.s003.pdf]

Removed Structures
Left-Lateral-Ventricle
Left-Inf-Lat-Vent
3rd-Ventricle
4th-Ventricle
Brain-Stem
CSF
Left-VentralDC
Left-vessel
Left-choroid-plexus
Right-Lateral-Ventricle
Right-Inf-Lat-Vent
Right-VentralDC
Right-vessel
Right-choroid-plexus
Optic-Chiasm
CC_Posterior
CC_Mid_Posterior
CC_Central
CC_Mid_Anterior
CC_Anterior
